# Supplementary material for: Neuropsychopharmacological Induction of (Lucid) Dreams: A Narrative Review
Source: Brain Sci. 2024 Apr 25;14(5):426. doi: 10.3390/brainsci14050426 (PMC11119155; doi:10.3390/brainsci14050426)
Supplement: Supplementary file 1 [file brainsci-14-00426-s001.zip › brainsci-2954069-supplementary.pdf]

Supplementary Materials

1. Sleep Regulation: Circadian and Homeostatic Processes

When photic stimuli from the environment enter the retina, peripheral oscillators that are molecularly controlled by hypothalamic structures synchronize and adapt to the endogenous rhythm of the organism, creating a rest-activity cycle of approximately 24 hours (circadian). The main structure involved in this process is the suprachiasmatic nucleus, which comprises two nuclei located bilaterally in the anterior hypothalamus and above the optic chiasm. In turn, the suprachiasmatic nucleus sends projections to structures that control several circadian physiological processes, through the expression of "clock genes" – circadian rhythmic genes that cyclically express themselves. Endogenous substances, such as adenosine [1], accumulate in proportion to the time awake, causing the homeostatic need for sleep, which is integrated with the circadian stimulus to initiate sleep, under the control of diencephalon structures.

The circadian input of the anterior hypothalamus and the homeostatic pressure of sleep activate the ventrolateral preoptic nucleus and median preoptic nucleus to initiate sleep, when gamma-aminobutyric acid (GABA) inhibits the acetylcholine (ACh), noradrenaline (NA), and serotonin (5-HT) arousal systems in the brainstem and also from the caudal hypothalamus [2]. The ventrolateral preoptic nucleus contains two types of sleep-active neurons: one population is excited by adenosine and may promote sleep in response to homeostatic sleep pressure, and the other population does not respond to adenosine but is inhibited by ACh, NA, and 5-HT, suggesting that it may help consolidate sleep once the arousal systems are inactive. The histaminergic system of the posterior hypothalamus and the cholinergic system of the basal forebrain are also inhibited, all of which are modulated by the orexinergic arousal system of the lateral hypothalamus.

Once sleep is initiated by the combined influence of homeostatic pressure and circadian propensity on the hypothalamic structures, prominent oscillatory rhythms can be detected in electroencephalographic recordings. These oscillations present as slow delta (~2Hz) and rapid spindle (~12Hz) rhythms, arising from the alternation between hyperpolarization and depolarization of intracortical neurons. This sleep stage is known as non-rapid-eye movement (NREM) sleep and is currently subdivided into three stages: N1, N2, and N3 [3,4].

When closing the eyes and reaching a state of mental relaxation, cortical alpha waves (8-12 Hz) are observed on electroencephalography, and are mostly replaced when initiating sleep. Stage 1 (N1) presents theta waves (4-8 Hz), lasting approximately a few minutes, which characterize a typical transitional state from waking to sleep. This stage may be accompanied by short dreaming, and/or hypnagogic hallucinations [5]. After the onset of sleep, stage 2 (N2), also known as light sleep, begins. The N2 sleep stage is characterized by the presence of spindles (11-14 Hz) and k-complexes (single delta waves, lasting approximately one second). It lasts for up to 50% of the total night of sleep. At this stage, it is also observed that the heart rate and body temperature decrease. Dreams can also happen, and they are mainly related to a simple thought, or to events that occurred during the day. Stage 3 (N3) is considered the deepest stage, with low frequencies (<4 Hz) and high delta wave amplitudes, which is why it is also commonly called "slow wave sleep". Dreams are less remembered during this deepest sleep stage.

Finally, the rapid-eye movement (REM) sleep stage has low voltage and high frequency activity, including theta (4-6 Hz) predominantly hippocampal, and gamma (30-90 Hz) in various areas such as the amygdala, hippocampus, thalamus and hypothalamus. With each cycle this stage becomes longer: the first cycle occurs approximately after 90 minutes of sleep onset and takes about 10 minutes, while the last cycle could take up to one hour. This stage is characterized by generalized muscle atony [4,6], due to glutamatergic neurons of the sublaterodorsal nucleus that excite neurons in the ventromedial medulla and spinal cord that produce GABA and glycine, which hyperpolarize motor neurons [7].

The regular alternation of NREM and REM sleep occurs with REM-on (cholinergic) cell groups promoting excitatory circuits and REM-off (aminergic) cell groups promoting inhibitory

circuits. Thus, REM sleep is potentiated cholinergically and suppressed aminergically. Activation of the brain during REM sleep results from the increased firing of reticular, thalamocortical, and cortical neurons, with the change in hippocampal activity from irregular rhythms to regular theta rhythms influenced by the brainstem and mediated by the medial septal nucleus of the basal forebrain, which is cholinergic [8,9].

ACh release in the cortex is similar during both wakefulness and REM sleep (and greater than that during NREM sleep), but differs in the basal forebrain, with greater activation during REM sleep [10]. Cholinergic neurons in the laterodorsal tegmental nucleus and pedunculopontine nucleus are maximally active during wakefulness and REM sleep, and selective optogenetic stimulation of cholinergic neurons in the laterodorsal tegmental nucleus or pedunculopontine nucleus promotes the transition from NREM sleep to REM sleep. REM-on cholinergic neurons project to the oral pontine nucleus, the "executive area" of REM sleep, and pharmacological stimulation of this area with cholinergic agonists promotes REM sleep [10]. In contrast, noradrenergic neurons in the locus coeruleus and serotonergic neurons in the dorsal raphe nucleus are active during wakefulness and almost cease firing during REM sleep.

Several lines of evidence indicate that, during wakefulness, these aminergic neurons inhibit REM-on cholinergic neurons in the laterodorsal tegmental nucleus and pedunculopontine nucleus, as well as neurons in the oral pontine nucleus [11]. Thus, NA and 5-HT neurons are activated during wakefulness, decrease their firing rate during slow-wave sleep, and stay nearly silent during REM sleep. Gottesmann [12] speculates that this decrease contributes to mental imagery during dreaming, which is primarily similar to symptoms of schizophrenia. Experimental deficits of NA during the wake state can impair emotional encoding by the amygdala and the hippocampus, as well as retrieval of mental processes, which could be compared to the REM sleep state (for review see [12]). One cluster of REM-on neurons is located dorsal and medial to the ventrolateral preoptic nucleus, and is called the extended ventrolateral preoptic nucleus. Neurons acting in this area, such as GABAergic/galaninergic neurons, which are active during REM sleep, may reduce REM sleep if the extended ventrolateral preoptic nucleus is damaged [13]. These cells innervate the dorsal raphe, locus coeruleus, and ventrolateral periaqueductal grey matter/ lateral pontine tegmentum, suggesting that the extended ventrolateral preoptic nucleus promotes REM sleep by inhibiting the brainstem neurons that suppress REM sleep.

During REM sleep, the activation of the nucleus accumbens is evidenced by the presence of neurons that are even more active than during wakefulness, implying that dopamine (DA) is probably released during REM sleep. This may be due to the inhibition of NA from the locus coeruleus, which are REM-off neurons, that modulate DA from ventral tegmental area [7]. In addition, DA uptake at NA terminals controls DA availability [14]. This could explain the delusions and hallucinations in dreams, or even the intensification of emotions observed by the high activation of the amygdala during REM sleep due to NA inhibition [12].

2. Mechanisms that Control REM Sleep and Dreaming

Solms [15] proposes that REM sleep and dreams are controlled by different mechanisms, since this sleep stage is modulated by cholinergic activity in the brainstem, and lesions in this area do not abolish dreams. This hypothesis suggests that dreaming is controlled by the forebrain, more specifically by dopaminergic neurons. Indeed, neuropsychological, radiological, and pharmacological findings suggest that the cholinergic mechanisms in the brainstem that control the REM state can only generate the psychological phenomena of dreams through the mediation of a second prosencephalic mechanism, probably dopaminergic [15]. Dreams can be manipulated by DA agonists and antagonists without concomitant changes in the frequency, duration, and density of REM, or even induced by focal stimulation of the forebrain during NREM when the involvement of REM mechanisms in the brainstem is excluded. Besides, evidence suggests that the mesocorticolimbic DA system plays a causal role in dream generation [15], since dreams are abolished in lesions of the frontal lobes, and this region, together with limbic structures (such as the amygdala and anterior cingulate), is interconnected with DA cells in the ventral tegmental area by a substantial number of fibers arising from the forebrain [16].

The Reward Activation Model proposes that the activation of the mesolimbic dopaminergic reward system during sleep contributes to memory processes, regulation of REM sleep, and the generation and motivational content of dreams [17]. In particular, it is speculated that the engagement of the mesolimbic dopaminergic reward system and associated limbic structures prioritizes information with high emotional or motivational relevance for (re)processing during sleep and dreaming, potentially allowing for adapted interactions with the environment, and therefore is critically important for individual survival. The mesolimbic dopaminergic reward system has been described as highly active during sleep, with activity in the ventral tegmental area during the end of NREM and mainly during REM sleep. There would be an increase in DA projection during REM periods to the hippocampus and pedunculopontine nucleus, as well as the amygdala and the suprachiasmatic nucleus. As mentioned before, the pedunculopontine nucleus is the site of REM-on neurons, and its increased activity during REM sleep is mediated through the DA neurons of the ventral tegmental area - which also modulates REM-off neurons of the locus coeruleus. DA neurons from the ventral tegmental area are part of the neuronal circuitry involved in active (or phasic) REM sleep. In addition, antagonizing NA into the ventral tegmental area significantly reduces waking and increases NREM sleep, without significantly affecting REM sleep. This suggests that under normal physiological conditions, NA inputs to the ventral tegmental area promote waking and reduce NREM sleep, and that this NA input to the ventral tegmental area could be from the locus coeruleus [7].

The Reward Activation Model supports that phasic DA signaling during REM sleep favors an offline reproduction of recent memory traces. These memories can serve as salient and novel stimuli to the pedunculopontine nucleus and ventral tegmental area, because relevant recent memories (e.g., emotional events, current concerns) are activated in the absence of associated contextual cues from wakefulness and cognitive control from the dorsolateral prefrontal cortex during REM sleep [17]. Glutamate and GABA are crucial in maintaining the sleep/wake cycle, as they are the main neurotransmitters in the central nervous system. GABA serves as the body's natural tranquilizer with around 40% of all neurons responding to it. GABA is also crucial for regulating attention and dreaming [18]. GABA in the pontine reticular formation promotes sleep, whereas glutamate is present at a higher concentration during REM than during NREM sleep. Evidence shows that even though cholinergic and monoaminergic neurons from the laterodorsal tegmental and pedunculopontine nucleus might be crucial for the maintenance of REM sleep, they are considered modulators [2]. On the other hand, glutamatergic sublaterodorsal neurons of the pons might be the central key for generating REM sleep. In addition, GABAergic neurons from the laterodorsal tegmental and pedunculopontine nucleus can fire both in wakefulness and REM sleep and to a lesser extent in NREM sleep [2]. GABA and glutamate have more diffuse and less discriminating projections, thus causing distinct patterns of activity in different areas, both with their antagonists and agonists. As an example, GABA REM-on neurons from the sublaterodorsal nucleus are connected with GABA REM-off neurons from the lateral pontine tegmentum and periaqueductal gray matter, proving the changes crucial for both REM and NREM regulation [19].

References

1. Reichert, C. F.; Deboer, T.; Landolt, H. P. Adenosine, caffeine, and sleep–wake regulation: state of the science and perspectives. *Journal of Sleep Research* **2022**, *31*(4). doi:10.1111/JSR.13597.

2. Scammell, T. E.; Arrigoni, E.; Lipton, J. O. Neural Circuitry of Wakefulness and Sleep. *Neuron* **2017**, *93*(4), 747–765. doi:10.1016/J.NEURON.2017.01.014.

3. Kaida, K.; Itaguchi, Y.; Iwaki, S. The AASM Manual for the Scoring of Sleep and Associated Events: Rules, Terminology, and Technical Specification. *(No Title)* **2007**, *12*(2). doi:10.1371/JOURNAL.PONE.0171907.

4. Adamantidis, A. R.; Gutierrez Herrera, C.; Gent, T. C. Oscillating circuitries in the sleeping brain. *Nature reviews. Neuroscience* **2019**, *20*(12), 746–762. doi:10.1038/S41583-019-0223-4.
5. Siclari, F.; Valli, K.; Arnulf, I. Dreams and nightmares in healthy adults and in patients with sleep and neurological disorders. *The Lancet Neurology* **2020**, *19*(10), 849–859. doi:10.1016/S1474-4422(20)30275-1.
6. Patel, A. K.; Reddy, V.; Shumway, K. R.; Araujo, J. F. Physiology, Sleep Stages. *StatPearls* **2022**.
7. Ratna, D.; Mondal, A. C.; Mallick, B. N. Modulation of dopamine from ventral tegmental area neurons by the LC-REM-OFF and PPT-REM-ON neurons in REMS regulation in freely moving rats. *Neuropharmacology* **2023**, *237*. doi:10.1016/J.NEUROPHARM.2023.109621.
8. Hobson, J. A. REM sleep and dreaming: towards a theory of protoconsciousness. *Nature reviews. Neuroscience* **2009**, *10*(11), 803–814. doi:10.1038/NRN2716.
9. Pace-Schott, E. F.; Hobson, J. A. The Neurobiology of Sleep: Genetics, cellular physiology and subcortical networks. *Nature Reviews Neuroscience 2002 3:8* **2002**, *3*(8), 591–605. doi:10.1038/nrn895.
10. Vazquez, J.; Baghdoyan, H. A. Basal forebrain acetylcholine release during REM sleep is significantly greater than during waking. *American journal of physiology. Regulatory, integrative and comparative physiology* **2001**, *280*(2). doi:10.1152/AJPREGU.2001.280.2.R598.
11. Vanini, G.; Torterolo, P. Sleep-Wake Neurobiology. In *Advances in Experimental Medicine and Biology*; Adv Exp Med Biol, 2021; Vol. 1297, pp 65–82. doi:10.1007/978-3-030-61663-2_5.
12. Gottesmann, C. The involvement of noradrenaline in rapid eye movement sleep mentation. *Frontiers in Neurology* **2011**, *DEC*. doi:10.3389/FNEUR.2011.00081/PDF.
13. Lu, J.; Bjorkum, A. A.; Xu, M.; Gaus, S. E.; Shiromani, P. J.; Saper, C. B. Selective activation of the extended ventrolateral preoptic nucleus during rapid eye movement sleep. *The Journal of Neuroscience: The Official Journal of the Society for Neuroscience* **2002**, *22*(11), 4568–4576. doi:10.1523/JNEUROSCI.22-11-04568.2002.
14. Carboni, E.; Silvagni, A. Dopamine reuptake by norepinephrine neurons: exception or rule? *Critical reviews in neurobiology* **2004**, *16*(1–2), 121–128. doi:10.1615/CRITREVNEUROBIOL.V16.I12.130.
15. Solms, M. Dreaming and REM sleep are controlled by different brain mechanisms. *The Behavioral and brain sciences* **2000**, *23*(6), 843–850. doi:10.1017/S0140525X00003988.
16. Mota-Rolim, S.; Ribeiro, S. Bases biológicas da atividade onírica; 2013; pp 201–227.
17. Perogamvros, L.; Baird, B.; Seibold, M.; Riedner, B.; Boly, M.; Tononi, G. The Phenomenal Contents and Neural Correlates of Spontaneous Thoughts across Wakefulness, NREM Sleep, and REM Sleep. *Journal of cognitive neuroscience* **2017**, *29*(10), 1766–1777. doi:10.1162/JOCN_A_01155.
18. Csiernik, R.; Pirie, M. A primer on sleeping, dreaming, and psychoactive agents. *Journal of Social Work Practice in the Addictions* **2023**, *23*(1), 3–23. doi:10.1080/1533256X.2022.2159641.
19. Gott, J. A.; Stücker, S.; Kanske, P.; Haaker, J.; Dresler, M. Acetylcholine and metacognition during sleep. *Consciousness and Cognition* **2024**, *117*, 103608. doi:10.1016/j.concog.2023.103608.
